# An Evaluation of Forced Distance Learning and Teaching Under Pandemic Conditions Using the Technology Acceptance Model

**DOI:** 10.3389/fpsyg.2021.701347

**Published:** 2021-10-21

**Authors:** Barbara Drueke, Verena Mainz, Martin Lemos, Markus Antonius Wirtz, Maren Boecker

**Affiliations:** ^1^Institute of Medical Psychology and Medical Sociology, University Hospital of RWTH Aachen University, RWTH Aachen University, Aachen, Germany; ^2^Audiovisual Media Center, Faculty of Medicine, RWTH Aachen University, Aachen, Germany; ^3^Department of Research Methods, University of Education, Freiburg, Germany

**Keywords:** technology acceptance model, TAM, COVID, acceptance, attitude, perceived usefulness (PU), perceived ease of use (PEOU)

## Abstract

**Research:** Due to the sudden outbreak of COVID-19 and the resulting pandemic situation, universities were forced to rapidly change their traditional pedagogical and didactical approach by shifting from mostly face-to-face teaching to entirely virtual and online teaching methods. Through this, a “forced” distance learning and teaching situation emerged. This study aimed at investigating the effect of these innovations on the implementation, acceptance, and use of the virtual teaching offer within the framework of the technology acceptance model (TAM).

**Methods:** A total of 218 students and 69 lecturers of a German Medical Faculty completed online questionnaires on the acceptance, satisfaction, and usefulness of the forced distance learning (FDL) and teaching (FDT), respectively. An extended version of the TAM was used to assess the acceptance of the students and lecturers of FDL and FDT. In order to estimate the multivariate dependencies, path analysis was employed using structural equation modeling (SEM).

**Results:** In general, students and lecturers reported being satisfied with the implementation of the FDL and FDT. Regarding the TAM model, the fit indices suggested an acceptable model fit for both groups. The model of the students revealed that the perceived usefulness had a strong predictive power on the attitude toward using and the perceived ease of use also predicted the attitude. The existing technical infrastructure as well as the general media affinity and pandemic-related worries proved to be positively associated with the perceived usefulness while data security worries and organization of online teaching predicted the perceived ease of use in students. The strong positive predictive power of the perceived usefulness for the attitude toward using was also evident in the model for the lecturers and the technical infrastructure predicted the perceived ease of use in the lecturers.

**Conclusion:** The TAM is a suitable framework to represent the implementation, acceptance, and use of the virtual teaching offer during the special pandemic situation at the university. However, personal and structural context factors were important predictors for the perceived usefulness and the perceived ease of use in the student group. The forced situation for learning and teaching makes it more difficult to predict the actual use of virtual teaching offers solely based on attitude.

## Introduction

The emergence of the internet and the development of modern technologies have affected education and learning. Still, traditional teaching and learning both take place in physical classrooms, while online learning is the exception in most universities irrespective of size, place, and time constraints (Liu et al., 2010). Due to the sudden outbreak of the disease COVID-19, German universities had to prepare an online/digital semester for their students as face-to-face teaching was restricted, and physical classrooms were not allowed [Hochschulrektorenkonferenz (HRK), 2020]. This had to be done under time pressure as the events required quick action. The situation challenged the educational systems in German universities and forced all involved persons to shift to an online mode of teaching overnight. The students and lecturers at RWTH Aachen University had to prepare for a digital semester in a very short time. The medical faculties of the university hospitals had a special role during the pandemic situation. In this study, the challenges of introducing digital teaching were particularly high since medical care had to be guaranteed during the time of the pandemic. Moreover, in addition to the theoretical content, studying medicine includes a large number of practical courses, the teaching of practical skills, hands-on exercises, and laboratory work that are difficult to teach or learn virtually. Because of these challenges, we were interested in the acceptance and satisfaction of the virtual teaching offer in the Medical Faculty of RWTH Aachen University during the summer term of 2020. Furthermore, we aimed at investigating both, students' as well as lecturers, potential acceptance or rejection of this offer and factors that might influence the implementation of digital learning and teaching, respectively. Since the students and lecturers had to prepare for a digital semester due to COVID-related restrictions, we referred to this matter as “forced distance teaching and learning” (FDT; FDL) in this study.

In this research, the proposed model is based on the technology acceptance model (TAM) as a framework to understand the role of acceptance and satisfaction with the virtual teaching method in this FDL and FDT situation. The TAM adapts the theory of reasoned action by Ajzen and Fishbein (1980) to explain the causal relationship between the internal beliefs (usefulness and ease of use), attitude, and computer usage behavior of the users (Davis, 1989). More recently, the TAM has been proven to be a robust model for predicting the acceptance of users when it comes to technology (Venkatesh and Davis, 1996, 2000; Venkatesh, 2000; Legris et al., 2003). Moreover, the TAM has attracted significant attention in e-learning research (Ahuja and Thatcher, 2005; Sumak et al., 2011; Shin and Kang, 2015). In its original form, the model assumes that the behavior of a person is determined by his or her voluntary attitude toward using a technology (ATU), which then results in a behavioral intention. In general, it is necessary to measure attitude and beliefs regarding the use of technology rather than attitude and beliefs directed toward the technology itself, since individuals might hold a positive view about the technology without being favorably disposed toward its use. Attitudes are formed from the beliefs a person holds about the use of a particular technology, which are seen as cognitive factors that might influence each other. The first belief “perceived usefulness” (PU) is the notion of the user which refers to their “subjective probability that using a specific application system will increase their job performance within an organizational context” (Davis, 1989, p. 320). The second belief “perceived ease of use” (PEOU) is “the degree to which the user expects the target system to be free of efforts” (Davis, 1989, p. 320). Furthermore, the model describes the influence of external variables, such as design features, that can affect the cognitive factors. These variables received attention in research as they could improve the understanding of how cognitive factors like PU and PEOU are formed or how they can be manipulated (Chin and Gopal, 1995). At a very early stage, Venkatesh and Davis (1996) further illustrated that the effectiveness of the model can be increased by such extensions. In a meta-analysis of Yousafzai et al. (2007), they classified various external variables into four categories of organizational-, system-, and personal characteristics of the users, among other variables. Other studies have rather focused on a few and specific factors, e.g., personal factors as playfulness (Moon and Kim, 2001; Estriegana et al., 2019) or learning styles (Al-Azawei et al., 2017). The study of Rauniar et al. (2014) found trustworthiness to be an important factor for the TAM model. Trustworthiness concerns the security of information posted on social media sites. For our purposes, we defined a similar construct which we called data security worries that assess worries regarding the misuse of data and worries of being spied on while using the video conferencing programs. Another particular interest for our study was the construct of the technical support as investigated by several researchers (Fathema et al., 2015; Servidio and Cronin, 2018). The study of Servidio and Cronin (2018) found that technical support, defined as interventions by technical staff to assist students in their usage, influences the usefulness and the ease of use. We defined our construct of technical infrastructure (TI) regarding aspects of good internet quality or suitable equipment, for example. Other studies (Persico et al., 2014; Fathema et al., 2015) further highlighted the importance of system components besides the availability of the technical infrastructure. System components deal with different issues concerning organizational aspects which are also of interest for the students in our study, i.e., organization of online teaching (OT). Researchers also focused on personal variables such as perceived self-efficacy (Fathema et al., 2015), indicating the judgment or the confidence of the own capability of the user when it comes to operating/navigating/working with a system. A related construct to this is the experience with technology as investigated by Sun and Zhang (2006). For our purposes, we defined a similar construct called general media affinity that indicates if somebody is an expert computer user. Finally and noteworthy, it has been shown that in mandatory environments, attitude strongly correlates with usage behavior (Leonard-Barton, 1998), and in our case, the pandemic is an extremely mandatory environment which is why we included pandemic-related worries into our model.

The main objective of the study was to define and empirically test a theory-based, extended TAM model. The focus was to see whether pandemic-specific conditions have an impact on the acceptance and perceived usefulness of virtual teaching. We hypothesized that certain personal and structural factors are more important in this forced COVID-19 situation compared with situations without pandemic conditions, for example, some technical equipment was not available for purchase. In our study, we proposed an extended TAM model including external variables that might impact the acceptance and usage of virtual teaching methods of the students and lecturers in this COVID-bound FDL and FDT situation. As proposed in the original model, we assumed that attitude toward using (ATU) should affect the actual system use (AU) in students and lecturers (ATU → AU). We also hypothesized that perceived usefulness (PU), as well as perceived ease of use (PEOU), have a strong effect on attitude toward using (PU → ATU, PEOU → ATU). Additionally, we hypothesized an association between ease of use and perceived usefulness (PEOU → PU). Furthermore, new relationships which were not proposed in the original model were established in this study to assume the person and context factors. The model considered the influence of personal and structural factors separately, supposing that these influence the attitude of a person toward using *via* PU and PEOU. We hypothesized that on the side of a person, general media affinity (GMA) and data security issues (DSW) would play a certain role and, above this, the characteristics of the special pandemic situation and the related subjective pandemic related worries (PW) should impact the model. More specifically, we hypothesized an effect of GMA on PU and PEOU (GMA → PU, GMA → PEOU). Similarly, data security worries (DSW) should affect PU and PEOU (DSW → PU, DSW → PEOU), while pandemic-related worries (PW) should predict PU more than PEOU (PW → PU). As external structural factors that might influence PU and PEOU, we assumed that the existing technical infrastructure (TI) and the organization of online teaching (OT) would play an important role assuming that those factors might have a greater predictive value for PEOU than for PU (TI → PEOU, OT → PEOU).

## Methods

### Participants

Among the human medicine (*N* = 1,300) and dentistry (*N* = 349) students contacted *via* the university mailing lists, 262 took part in the study, and 218 (13.2% of *N* = 1,649) completed the questionnaire. Participants did not receive any financial compensation for their participation. Only those participants who completed the online survey and actively send it off were included in the analysis. Exactly 159 (73%) of the students with completely answered questionnaires were women. The majority of students participating in the study was still in the preclinical phase of the medical studies (2nd = 28.4%; 4th = 22.9%, 6th = 22.9%, 8th = 9.2% and 10th = 12.8%). Students reported using the virtual teaching offer on an average of 15 h per week (Monday–Friday) (*M* = 15, *SD* = 10) and an average of 5 (*M* = 5, *SD* = 5) h on weekends. Among the students, 25% had reported working in health care due to additional demands during the COVID-19 pandemic. From all of the professors and scientists of the Faculty of Medicine, about 300 persons were involved in teaching during the summer term of 2020. There were 260 lecturers who made videos for their teaching to be available, from whom, 106 (40.8%) took part in the study and 69 (26.5%) completed the questionnaire. Among the lecturers, 32 (46.4%) with completely answered questionnaires were women. The majority of lecturers were between 30 and 60 years of age (age groups: 30–40 = 33.3%; 41–50 = 20.3%, 51–60 = 28.9%, 17.4% were younger or older). On average, lecturers have been teaching for an average of 13 years (*M* = 13, *SD* = 10). Across the semester, the lecturers taught on average 20 h (*M* = 20, *SD* = 17). The reorganization of the teaching including the familiarization with the new technique resulted in an additional workload of on average of 9 h for this semester (*M* = 9, *SD* = 13). Respondents at 52% indicated that their primary activity would be research in addition to teaching, while 30% reported mainly work in health care. Only 15 lecturers indicated to have had experience with online teaching in former semesters.

### Data Collection

The study was carried out at the Medical Faculty of RWTH Aachen University at the end of the summer term of 2020. We collected data in July 2020 through an online survey using the program Limesurvey GmbH (2020) (Lime Survey, Hamburg, Germany). The study was approved by the Ethics Committee of the Medical Faculty of RWTH Aachen University (EK 227/20). In the introductory section of the online survey, we provided information about the study, i.e., justification, aim, and methods, and the permission of the participants to withdraw at any point. We also explained how we were going to safeguard anonymity and confidentiality. All data were treated according to the European legislation on data protection. Participants accepted voluntary participation before completing the online survey by ticking a box that stated, “I have read and understood the above information and agree voluntarily, to participate in this survey by clicking on NEXT. I am aware that I can cancel the survey at any time.”

### Survey Instruments

Two online questionnaires were generated, one for students and one for lecturers (as shown in Supplementary Table 2). It was tried to construct both questionnaires as similar as possible. However, since the student version assessed the FDL situation and the lecturer version the FDT situation, they both differed slightly from each other in some subscales regarding content and the number of items. For this reason, data for students and lecturers were analyzed separately.

In both versions, participants were first asked to provide some demographic information, e.g., age, gender, occupation. In the following section, participants had to complete questions as indicators for the original TAM subscales (PU and PEOU), ATU, and AU. Additionally, three-person and two structural context variables were collected. The person context factors included questions on GMA in assessing whether one is a skilled computer user who easily familiarizes with new software, on DSW in assessing worries regarding misuse of data and worries of being spied on while using the video conferencing programs, and on PW in assessing whether one is negatively affected by the COVID-19 pandemic. The structural context factors included questions on the TI, such as whether one has suitable technical equipment and good Internet quality, and the organization of OT (only in student version) in assessing the structural organization of the OT. Regarding the survey, participants were instructed to refer to the live streams and video recordings of lectures and/or seminars in their answers. The TAM items, as well as personal and structural context variables assumed for the extended TAM model, were assessed using a 4-point Likert scale (Likert, 1932) ranging from “1 = do not agree at all” to “4 = fully agree,” indicating to what extent participants agreed with the respective statements. Overall, the FDL-version for the students consisted of 29 items, while the FDT-version for the lecturers of 22 items and took about 15 min to complete.

### Item Generation

As outlined above, the survey instrument consisted of the four original TAM model factors which are PU, PEOU, ATU, and AU, three subscales assessing the person context factors which are GMA, DSW, and PW, as well as two subscales assessing the structural context factors which are TI and OT, while the latter subscale was only included in the FDL-version for the students. These structural and person variables were considered as potential influencing factors regarding the acceptance of FDL and FDT as judged by a five-headed expert team and five students, respectively. The items for the original TAM model factors (Davis, 1989) were adapted to the FDL- and FDT-situation. The items for all other subscales were newly formulated. Easily understood language was used to prevent ambiguous statements and to help minimize errors due to misleading expressions. The questionnaire was revised by several experts to determine whether the questions were appropriate and confirm that the statements were unambiguous. The items can be seen in the Supplementary Material.

### Data Analysis

For the descriptive statistics of the scales, SPSS 25 for Windows software (SPSS Inc., Chicago, IL, USA) was used. Means (*M*) and *SD*s for variables incorporated in subsequent analyses and their intercorrelations were calculated. The intercorrelations can be found in the Supplementary Table 2. Before using structural equation modeling (SEM) (Bollen, 1989; Little and Kline, 2016), the reliabilities of the subscales were determined using Cronbach's Alpha and the dimensional structure was investigated using confirmatory factor analysis (CFA). For Cronbach's Alpha, values ≥0.70 indicate acceptable reliability. The item was deleted, if single subscale items showed low item-total correlations (<0.4) and Cronbach's Alpha could be improved when deleting the respective item. The 9-factorial CFA was only calculated for the students because of the small sample size of the lecturers.

The analysis aimed to estimate unbiased latent model parameters for the TAM models specifications. Estimating the structural model and the measurement models simultaneously is generally a valid approach to yield unbiased estimates. In a single analysis step, systematic variance components and error variance components are estimated for each construct (Bollen, 1989; Little and Kline, 2016). Only the systematic variance components are considered when modeling construct associations. This is equivalent to the mitigation-corrected parameter estimation (Steyer and Eid, 2002). However, this approach is not stably applicable for the data set of lecturers due to the too small sample size (*N* = 69) and the high number of parameters to be estimated (*N* = 56) (Schermelleh-Engel and Moosbrugger, 2003).

The modeling approach of Sass and Smith (2006) allows the determination of the reliability-corrected latent correlations and regression coefficients, although the number of parameters to be estimated is considerably reduced. This approach consists of two steps:

First, the reliability of the constructed assessment must be determined. For each construct, the latent trait and the reflective indicators in the measurement model are defined. This corresponds to the CFA of the constructs. Based on the estimated standardized model parameters (γ_i_ = factor loading; θ_ii_ = measurement error variances), construct-specific composite reliability values (Reuterberg and Gustafsson, 1992) can then be determined:


$$\begin{array}{l} CR\;=\;\frac{{\left(\sum_{i=1}^{c}\gamma_{i}\right)}^{2}}{{\left(\sum_{i=1}^{c}\gamma_{i}\right)}^{2}+\sum_{i=1}^{c}\theta_{i}} \end{array}$$

Second, in the final estimation of the comprehensive model, only one indicator is used for each construct, namely the scale value (mean value over the indicator items of the original measurement model). The error variance of each construct is fixed to the value [(1–CR)^*^variance (scale value)].

This procedure ensures that the reliability correction is based on the same assumptions as in the simultaneous estimation of structural and measurement models (assuming at least congeneric measurements) but addresses a considerably more parsimonious model structure in the final estimation.

Since only one item for the construct PW was answered by the lecturers, reliability was estimated based on the three student items. The Spearman-Brown correction formula was applied to estimate the reliability of a single item for the students. This value was adopted as the reliability estimate for the instructor item.

Model fit was evaluated using measures of absolute model fit, e.g., root mean square error of approximation (RMSEA), and measures of incremental fit, e.g., Tucker-Lewis index (TLI), comparative fit index (CFI). The RMSEA indicates the proportion of variance-covariance information which is not correctly predicted by the model. As a criterion of acceptable fit, values of ≤ 0.08 or ≤ 0.05 are deemed as indicating an acceptable or good fit. The same applies to the standardized root mean square residuals (SRMR). In addition, the TLI and the comparative fit index CFI were calculated as measures of the incremental model fit. For these measures, values ≥0.90 (Hu and Bentler, 1998) or ≥0.95 (Little and Kline, 2016) are suggested to indicate an acceptable model fit. The maximum likelihood estimation procedure (Little and Kline, 2016) implemented in the software AMOS 26 (IBM, Armond, New York, USA; Arbuckle and Wothke, 1999) was used to estimate the model parameters. As for the CFA, the same procedure (Little and Kline, 2016) implemented in the same software (Arbuckle and Wothke, 1999) was used to test the structural models. The input for SEM was the empirical covariance matrix. To accept a theory-based specified SEM as a plausible explanatory model for the empirical data, measures of absolute model fit, e.g., non-significant *X*^2^, RMSEA, SRMR, and measures of incremental fit, e.g. TLI, CFI, were calculated. In case of insufficient model-fit potential sources of the model, the violation was analyzed by inspecting unexplained residual correlations, i.e., modification indices, as well as insufficient indicator-construct associations, i.e., indicator reliabilities.

Indicators of local fit for the latent variables assess whether constructs can be reliably estimated from their indicators. Recommended thresholds were used to determine a good local model fit: Average Variance Extracted (AVE) ≥0.5, factor reliability ≥0.6, reliability (Cronbach's Alpha) ≥0.7, and Residual-Correlations ( ≤ 0.3). Indicator reliabilities should exceed the value of 0.4 to ensure that each item is sufficiently associated with the assumed underlying latent variable (Little and Kline, 2016).

Data is available: https://osf.io/r97ha/.

## Results

For some subscales, items were removed due to weak item-construct associations, i.e., low indicator reliabilities. Thus, one item each had to be removed from PU, AU, GMA, PW, and TI in the student version. In the version of the lecturers, one item each was removed for AU, GMA, and TI. All of the remaining items had acceptable indicator reliability. An overview of all items with their corresponding indicator reliabilities as well as an English translation can be found in Supplementary Table 2.

The final number of items in each subscale and the descriptive statistics for the students and the lecturers can be found in Table 1.

**Table 1:** Descriptive statistics for the extended TAM model.

**Factors and predictors**	**Scale**	**Students**										**Lecturers**								
		**N_**items**_**	**α**	**CR**	**AVE**	**M_**i**_ (SD)**	**M_**S**_ (SD)**	**SKEW**	**Kurtosis**	**VAR_**SV**_**	**FeV**	**N_**items**_**	**α**	**CR**	**M_**i**_ (SD)**	**M_**S**_ (SD)**	**SKEW**	**Kurtosis**	**VAR_**SV**_**	**FeV**
TAM factors	PU	3	0.89	0.89	0.73	3.20 (0.78)	9.61 (2.33)	−0.79	−0.07	0.60	0.07	3	0.78	0.78	2.97 (0.60)	8.91 (1.80)	0.02	−0.42	0.35	0.08
	PEOU	2	0.70	0.70	0.54	3.43 (0.70)	6.86 (1.40)	−1.11	1.86	0.59	0.08	2	0.51	0.52	3.13 (0.62)	6.26 (1.23)	−0.23	−0.81	0.55	0.07
	ATU	4	0.87	0.87	0.64	3.13 (0.77)	12.52 (3.09)	−0.95	0.38	0.48	0.14	4	0.86	0.87	2.61 (0.75)	10.43 (2.99)	−0.11	−0.76	0.37	0.18
	AU	2	0.64	0.64	0.47	3.63 (0.54)	7.26 (1.08)	−1.11	0.68	0.29	0.10	2	0.69	0.69	2.78 (0.86)	5.57 (1.72)	−0.42	−0.51	0.73	0.23
Person	PW	3	0.66	0.68	0.44	2.24 (0.73)	6.71 (2.19)	0.56	−0.05	0.53	0.17	1	–	(0.42)	2.62 (0.82)	2.62 (0.82)	0.16	−0.63	0.67	0.39
	GMA	2	0.75	0.76	0.61	3.04 (0.77)	6.08 (1.54)	−0.42	−0.54	0.59	0.14	2	0.75	0.74	3.22 (0.59)	6.45 (1.18)	−0.23	−0.57	0.34	0.09
	DSW	2	0.87	0.87	0.78	1.71 (0.74)	3.42 (1.47)	1.44	1.05	0.54	0.07	3	0.85	0.85	2.37 (0.86)	7.10 (2.58)	0.18	−1.02	0.73	0.11
Structure	TI	2	0.58	0.59	0.43	3.41 (0.62)	6.82 (1.24)	−0.69	0.67	0.38	0.16	2	0.64	0.64	3.15 (0.66)	6.30 (1.32)	−0.27	−0.79	0.43	0.15
	OT	4	0.66	0.68	0.35	2.72 (0.58)	10.86 (2.33)	−0.19	−0.32	0.34	0.11	–	–	–	–	–	–	–	–	–

PU, perceived usefulness; PEOU, perceived ease of use; ATU, attitude toward using; AU, actual system use; PW, pandemic related worries; GMA, general media affinity; DSW, data security worries; TI, technical infrastructure; OT, organization of online teaching; N_items_, number of items; α, Cronbach's α; CR, composite reliability; AVE, average variance extracted; M_i_, mean of scale items; SD, standard deviation; M_S_, scale mean; SKEW, skewness; VAR_SV_, variance of the scale value; FeV, Fixed error variance: (1-CR)*VAR.

### Descriptive Statistics for the Students

The actual system use (AU) proved to be high from the student perspective with the item mean across the subscale items being (*M* = 3.63, *SD* = 0.54). The perceived usefulness (PU, *M* = 3.2, *SD* = 0.78) and the perceived ease of use (PEOU, *M* = 3.4*, S*D = 0.70) were considered high and the attitude of willingness toward using (ATU, M = 3.1, SD = 0.77) was indicated as well. Substantially lower values prevailed for the pandemic related worries (PW, *M* = 2.24, *SD* = 0.73), the worries regarding data security (DSW, M = 1.7, SD = 0.74), and the organization of OT (OT, *M* = 2.7, *SD* = 0.58). The general media affinity (GMA, *M* = 3, *SD* = 0.77) and the availability of technical infrastructure (TI, *M* = 3.4, *SD* = 0.62) were evaluated from the perspective of the students as high, i.e., students reported to quickly find their way around computers and to have good technical equipment. Overall, the students reported a high level of satisfaction and acceptance with relatively low concerns about the pandemic and data security.

### Descriptive Statistics for the Lecturers

Overall, for the lecturers, most means across subscale items were slightly lower than the means found in students, but values for PEOU (*M* = 3.13, SD = 0.62) and PU (*M* = 2.97, *SD* = 0.6) proved to be high indicating an overall strong agreement in the lecturers as well. For ATU (*M* = *2.61*, SD = 0.75) and AU (*M* = 2.78*, SD* = 0.86), lecturers reported a slightly lower but still positive agreement. Compared with the means of the student group, PW (*M* = 2.62, *SD* = 0.82) and DSW were slightly higher (*M* = 2.37, *SD* = 0.86) indicating a moderate level of worries, i.e., lecturers were more worried about their data security and had more pandemic related worries than the students indicated. The TI was also rated as high (*M* = 3.15, *SD* = 0.66).

### Analysis of Latent Structural Path Model for the Students

The confirmatory factor analysis revealed acceptable global model fit (χ^2^ = 430.99, df = 219, *p* < 0.01, RMSEA = 0.067, TLI = 0.89, CFI = 0.91, SRMR = 0.06).

**Figure 1:**
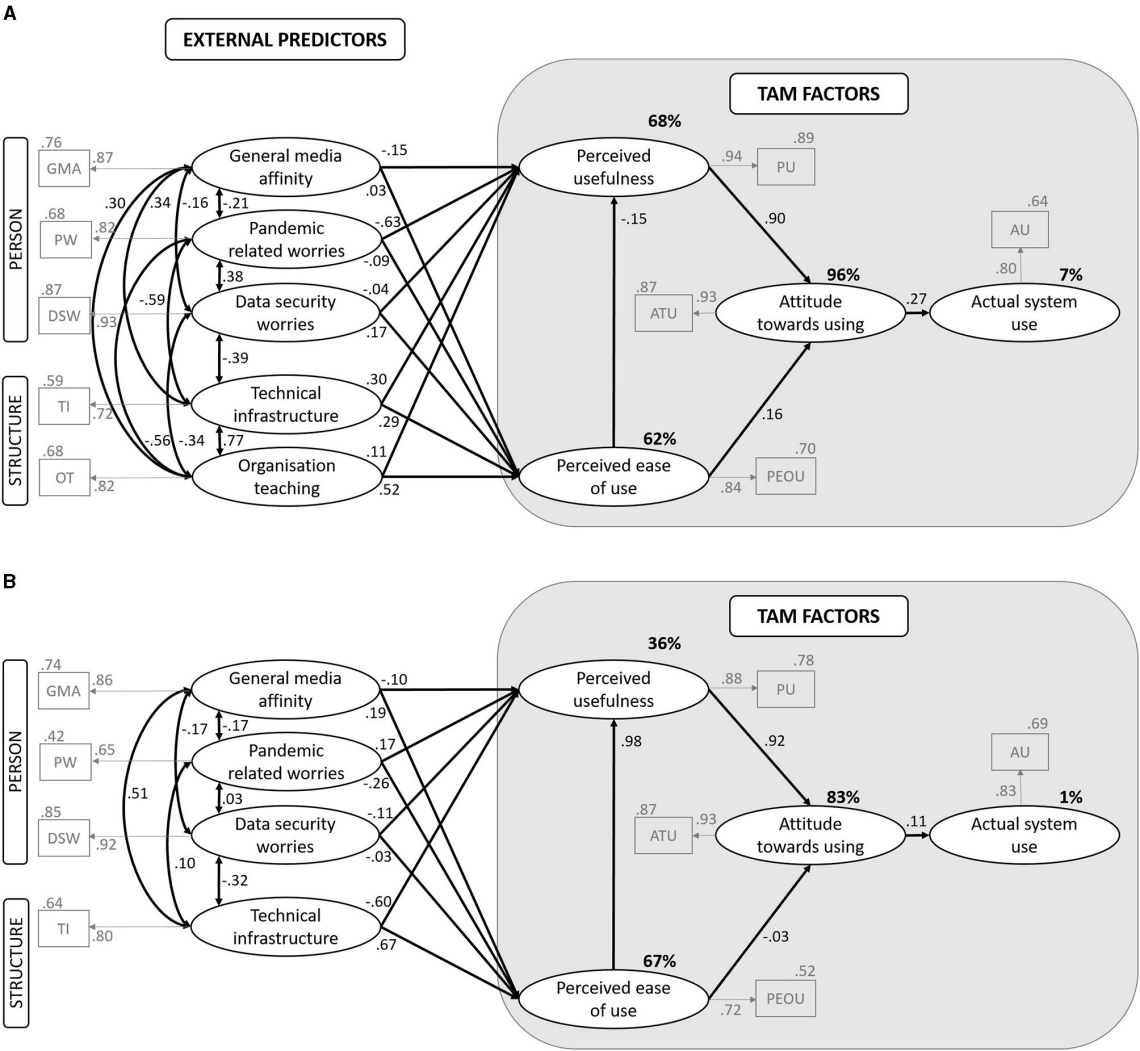
Latent structural path models. **(A)** Students. **(B)** Lecturers. Rectangles indicate observed indicator variables. Ovals indicate latent variables. Numbers on arrows indicate standardized regression weights.

Testing the latent structural path model for students (as shown in Figure 1A), we found a valid model (χ^2^ = 28.49, df = 12, *p* = 0.005, RMSEA = 0.08, TLI = 0.93, CFI = 0.98, SRMR = 0.035). 89% of variance in the PU and 62% of the variance in the PEOU could be explained by the external factors. Furthermore, 96% of variance in the ATU could be explained by the model factors. In contrast to that, only 7% of variance in the AU was explained by the model factors. The GMA showed a negative predictive value for the PU (β = −0.15, *p* = 0.04). The technical infrastructure (TI, β = 0.3, *p* = 0.09) also predicted the PU, but the best predictor for the PU were the pandemic related worries (PW, β = −0.63, *p* < 0.001). The organization of OT (β = 0.52, *p* =. 01) has a high predictive value for the PEOU and the DSW also predicted the PEOU (β = 0.17 *p* = 0.05). The ATU was highly predicted by the PU (β = 0.90, *p* < 0.001), but also by the PEOU (β = 0.16, *p* = 0.002). The ATU has a small but predictive value for the AU (β = 0.27, *p* = 0.002).

Regarding the indicators of local fit the factor reliabilities were acceptable and are shown in Table 1. The indicator reliabilities were ≥0.4 for most of the items, but six out of 24 items showed values below this threshold (as shown in Supplementary Table 2). However, these items were kept in order to improve subscale reliability.

### Analysis of the Latent Structural Path Model for the Lecturers

For the lecturers, we found an acceptable, non-significant model fit: χ^2^ = 16.43 df = 10, *p* = 0.08, RMSEA = 0.1, CFI = 0.92, TLI = 0.79, SRMR = 0.069. 67% of the variance in the PEO) could be explained by the model factors while only 36% of the variance in the PU. Furthermore, 83% of the variance in the ATU could be explained by the model factors, while almost no variance (1%) in the AU could be explained by the ATU. The PU has a really strong predictive value for the ATU (β = 0.92, *p* < 0.001). Different than expected, the PEOU has almost no predictive value for the ATU (β = 0.08, *p* > 0.05). The PEOU was best predicted by the TI (β = 0.67, *p* = 0.02). Different from the student sample, there was no effect of the ATU on the AU (β = 0.11, *p* > 0.05), indicating that the attitude did not provide predictive value for the actual use in the lecturer group. As in the student group, the PEOU has no significant impact on the PU (β = 0.97, *p* > 0.05). Different from the student group, TI did not show a predictive value for PU (β = −0.61, *p* > 0.05). In both cases, we saw high estimates which might indicate that the PEOU could predict the PU, and TI could predict PU, respectively. The lack of significance could be explained by the high standard errors at the same time which might be due to the small sample size of the lecturers. For the lecturers, neither the DSW, PW, nor GM provided any predictive value for the PU or the PEOU (as shown in Figure 1B).

## Discussion

In this study, an extended TAM model was used to investigate the acceptance and usage of the students and lecturers of the online teaching and learning offered at a Medical Faculty of a German university in 2020. The students and lecturers were in a very special situation as they were forced to learn and teach with the help of online tools as face-to-face-teaching and -participation was not allowed due to the risk of infection with COVID-19. Therefore, we were interested in finding out whether an extended TAM model holds up under these forced conditions and which factors are particularly relevant. The extended TAM model assumed the importance of different external factors influencing the acceptance and usage of the students and lecturers of online learning and teaching, respectively. The TAM model was extended by the personal variables which are PW, GMA, and DSW, as well as the structural context variables TI and OT. Indeed, this is important as Legris et al. (2003) stated that other variables should be included to understand those factors that affect technology adoption. This conclusion was confirmed by Edmunds et al. (2012) to indicate that the two factors, namely ease of use and usefulness, may not identify all significant components in predicting technology acceptance. The study of Holden and Karsh (2010) also stated in their review that an important future direction for TAM is to adapt the model specifically to the context, which is in our case the COVID-19 pandemic.

Overall, the implementation of digital learning and teaching has received positive resonance from both students and teachers. The high means for PU and ATU indicated that students and teachers conveyed a high level of satisfaction with the digital teaching offer. Furthermore, both groups did not report high levels of worries either for pandemic-related worries or for worries on data security. This meant that on average, the worries and the associated burden seemed manageable in both groups. AU was reported to be very high for the students indicating that the students not only assessed the digital learning offer as useful but also actually used it. The high mean values for TI also showed that both groups had suitable technical equipment for using the learning/teaching offer which is an essential prerequisite for acceptance and usage of digital learning and teaching.

An extended TAM was found to be an appropriate model to investigate the effects of the COVID-19 pandemic on the acceptance and usage of virtual learning and teaching for students. The comparison of models showed some differences between the models of the lecturers and the students. Furthermore, the models also differed in several aspects as the former is about learning and the latter about teaching. For the students, the aspect of the organization of online teaching, i.e., When does a course start? Where can I find the information about the link to the online course?, was very relevant, while these points were rather obligatory on side of the lecturers and in their responsibility regardless of the pandemic situation. Concerning the aspect of pandemic-related burden, the student model also differed from the model of the lecturers. In comparison with the lecturers, students might, for example, not be able to pursue employment during a lock-down, which might result in financial difficulties.

So far, the original model assumed that the PU and PEOU predict ATU, which was found in several studies (Holden and Karsh, 2010; Fathema et al., 2015). ATU in turn should predict AU. In the group of students, the PU and the PEOU were confirmed as predictors of ATU. This supported our hypothesis and was consistent with the results from the literature (Wong et al., 2013). The TI significantly predicted the PU but not PEOU. This indicated that good technical equipment has a predictive value for the perceived usefulness of students. The empirical evidence of the importance of external variables like TI the TAM has been found in the past (for example, see Yeou, 2016; Servidio and Cronin, 2018). The ATU was confirmed as a predictor of the AU which is in line with our hypotheses. This effect was found but not as high as in other studies which could be due to the forced situation. The PEOU was best predicted by the OT. The best negative predictor for the PU was the PW indicating that students who felt burdened by the Corona-pandemic reported lower perceived usefulness which could be due to the stress of the overall situation. This is also in line with our hypotheses. Interestingly, the PW was negatively correlated with the TI and OT indicating that students with good technical equipment stated that they were less stressed by the pandemic and were able to cope well with organizational aspects (see Figure 1A). TI was also negatively correlated with DSW which indicated that students with good technical equipment reported fewer worries on data security. The DSW was also significantly correlated with the PW indicating that the pandemic was a stressful situation with lots of concerns to the students at all. Interestingly and contrary to our hypotheses, the PEOU had no predictive value for the PU. This result could be explained by the fact of a rather forced distance learning situation. There was 68% of the variance in the PU, 62% in the PEOU, and 96% in the ATU that could be clarified. Only 7% of the variance in the AU could be explained by the model factors which might be a result of the forced situation as well in which students had almost no alternative than using the digital learning offer.

The predictive power of PU on ATU was also evident in lecturers which is in line with evidence from the literature (Holden and Karsh, 2010; Fathema et al., 2015). The TI failed to reach significance for the PU which might be due to a high estimation error. Compared with the student group and contrary to our hypothesis, the PEOU was not confirmed as a predictor for the ATU, and the ATU in turn has no predictive power for the AU. This might be due to the pandemic situation in which lecturers had *de facto* no other way to reach their students than *via* the digital offering. This emphasized the forced part of teaching even more. Interestingly, we found an impact of the TI on the PEOU in the lecturers emphasizing the importance of good equipment and indicating that lecturers with good technical equipment perceived the challenges in the implementation as simple or easy to use. For the lecturers, there was also a positive correlation between the TI and GMA (as shown in Figure 1B) which shows that lecturers with a high affinity for media were well equipped. For the lecturers, we found no significant effect for the PEOU on the PU. The influence of the PW on the PU was not evident in the lecturers. This may be because PW was seen independently of the usefulness. For the students, we found a relationship between the PW and the TI which was not evident in lecturers as well. There was 36% of the variance in the PU and 67% in the PEOU that could be explained by personal and structural context factors in the model. Furthermore, 83% of the variance in the ATU could be explained by PU and PEOU, but almost no variance could be explained in the AU (1%). In the model of the lecturers, the explanation of variance was lower which could be due to the not optimal model fit and small sample size in lecturers. All in all, for the lecturers many paths did not reach significance and we did not find as much evidence for our hypotheses as for the students.

The following limitations must be considered when interpreting the study results. The data and the resulting SEM originated from a study with a cross-sectional design which did not allow a causal interpretation of the relationships found in the predictive model. The cross-sectional design was mainly because the sudden outbreak of COVID-19 meant that everything had to be rearranged under enormous time pressure. In the future, there should be the conduction of model-based intervention trials to gain enhanced evidence. Although the model postulates causal effects, these cannot be proven using the analytical approach. We only estimated the strength of the effects, assuming the model structure. But whether these assumptions were appropriate cannot be tested empirically. One limitation is the model fit, which was not optimal in all parameters, especially for the lecturers. But with the given preconditions, we presented the best possible result. Another limitation is the relatively small sample size, especially for the lecturers. The small sample size may explain why some results and indices did not become significant due to insufficient test power. Nevertheless, a sample size >47 generally allowed the detection of medium effects sizes with sufficient power (1–β <0.2; Faul et al., 2009). Several explanations are conceivable. On the one hand, it is possible that some courses were not offered online as they required face-to-face interaction or the use of special equipment, for example. On the other hand, the study participation was voluntary, and there was no payment for the participants. Furthermore, data collection took place during the exam period which might have reduced the number of participants. Another important point to mention is that the role of surveys in seeking information is problematic as low response rates are common (for example, see Grava-Gubins and Scott, 2008). Researchers need to investigate alternative strategies for achieving higher rates of response, especially as response rates were found to be lower for digital invitations compared with paper-based invitations (Ebert et al., 2018).

Another limitation is ceiling effects for some measures, e.g., the mean of the AU scale, resulting in likely rather small variances that limit the potential to identify substantial effects. This is not surprising and probably due to the special pandemic situation as all participants were in a forced situation in which there was no alternative to online learning/teaching.

All in all, during the first lockdown in Germany, we assessed the implementation, acceptance, and use of the virtual teaching offer at a German university. The results showed that an extended TAM is a suitable framework to test for this. The PU strongly predicted the ATU of students and of lecturers while the influence of the PEOU seemed to be smaller in a pandemic situation in which all participants were forced to use online learning and teaching, respectively. External variables like PW strongly predicted the PU especially for students, while the TI was an important predictor for the ease of use in both groups. However, the forced situation for learning and teaching made it more difficult to predict the actual use of virtual teaching offers based on attitude.

## Data Availability Statement

The raw data supporting the conclusions of this article will be made available by the authors, without undue reservation.

## Ethics Statement

The studies involving human participants were reviewed and approved by Ethics Committee of the Medical Faculty of RWTH Aachen University (EK 227/20). Written informed consent for participation was not required for this study in accordance with the national legislation and the institutional requirements.

## Author Contributions

BD: conceptualization, data curation, formal analysis, investigation, methodology, visualization, and writing—original draft. MW: conceptualization, data curation, formal analysis, methodology, and writing—review and editing. VM: conceptualization, methodology, data curation, formal analysis, validation, and writing—review and editing. ML: conceptualization, project administration, methodology, resources, and writing—review and editing. MB: conceptualization, formal analysis, data curation, methodology, project administration, and writing—review and editing. All authors contributed to the article and approved the submitted version.

## Conflict of Interest

The authors declare that the research was conducted in the absence of any commercial or financial relationships that could be construed as a potential conflict of interest.

## Publisher's Note

All claims expressed in this article are solely those of the authors and do not necessarily represent those of their affiliated organizations, or those of the publisher, the editors and the reviewers. Any product that may be evaluated in this article, or claim that may be made by its manufacturer, is not guaranteed or endorsed by the publisher.

## References

[B1] Ahuja M. K. Thatcher J. B. (2005). Moving beyond intentions and toward the theory of trying: effects of work environment and gender on post-adoption information technology use. Manage. Inf. Syst. Q. 29, 427–459. 10.2307/25148691

[B2] Ajzen I. Fishbein M. (1980). Understanding Attitudes and Predicting Social Behavior. Englewood Cliffs, NJ: Prentice-Hall.

[B3] Al-Azawei A. Parslow P. Lundqvist K. (2017). Investigating the effect of learning styles in a blended e-learning system: an extension of the technology acceptance model (TAM). Aust. J. Educ. Technol. 33, 1–23. 10.14742/ajet.2741

[B4] Arbuckle J. L. and Wothke, W. (1999). AMOS 4.0 User's Guide. Chicago, IL: Small-Waters Corporation

[B5] Bollen K. A. (1989). Structural Equations with Latent Variables. New York, NY: Wiley. 10.1002/9781118619179

[B6] Chin W. W. Gopal A. (1995). Adoption intention in GGS: relative importance of beliefs. Data Base Adv. 26, 42–63. 10.1145/217278.217285

[B7] Davis F. (1989). Perceived usefulness, perceived ease of use, and user acceptance of information technology. Manage. Inf. Syst. Q. 13, 319–40. 10.2307/249008

[B8] Ebert J. F. Huibers L. Christensen B. Christensen M. B. (2018). Paper- or web-based questionnaire invitations as a methods for data collection: cross-sectional comparative study of differences in response rate, completeness of data, and financial cost. J. Med. Internet Res. 20:e24. 10.2196/jmir.8353 29362206 PMC5801515

[B9] Edmunds R. Thorpe M. Conole G. (2012). Student attitudes towards and the use of ICT in course study, work and social activity: a technology acceptance approach. Br. J. Educ. Technol. 43, 71–84. 10.1111/j.1467-8535.2010.01142.x

[B10] Estriegana R. Medina-Merodio J. A. Barchino R. (2019). Student acceptance of virtual laboratory and practical work: an extension of the technology acceptance model. Comput. Educ. 135, 1–14. 10.1016/j.compedu.2019.02.010

[B11] Fathema N. Shannon D. Ross M. (2015). Expanding the technology acceptance model (TAM) to examine faculty use of learning management systems (LMSs) in higher education institutions. J. Online Learn. Teach 11, 210–232. Available online at: https://jolt.merlot.org/Vol11no2/Fathema_0615.pdf

[B12] Faul F. Erdfelder E. Buchner A. Lang A.-G. (2009). Statistical power analyses using G^*^Power 3.1: tests for correlation and regression analyses. Behav. Res. Methods 41, 1149–1160. 10.3758/BRM.41.4.1149 19897823

[B13] Grava-Gubins I. Scott S. (2008). Effects of various methodologic strategies: survey response rates among Canadian physicians and physicians-in-training. Can. Family Phys. 54, 1424–1430. 18854472 PMC2567275

[B14] Hochschulrektorenkonferenz (HRK) (2020). German rectors Conference (2020). Bestimmung im Sommersemester 2020. Available online at: https://www.hrk.de/themen/hochschulsystem/covid-19-pandemie-und-die-hochschulen/ (accessed November 10, 2020).

[B15] Holden R. J. Karsh B. T. (2010). The technology acceptance model: its past and its future in health care. J. Biomed. Inform. 43, 159–172. 10.1016/j.jbi.2009.07.002 19615467 PMC2814963

[B16] Hu L. Bentler P. M. (1998). Fit indices in covariance structural equation modeling: sensitivity to underparameterized model misspecification. Psychol. Methods 3, 424–453. 10.1037/1082-989X.3.4.424

[B17] Legris P. Ingram J. Collerette P. (2003). Why do people use information technology? A critical review of the technology acceptance model. Inf. Manage. 40, 191–204. 10.1016/S0378-7206(01)00143-4

[B18] Leonard-Barton D. (1998). Implementation characteristics of organizational innovations. Commun. Res. 15, 603–631. 10.1177/009365088015005006

[B19] Likert R. (1932). A technique for the measurement of attitudes. Arch. Psychol. 22, 140–55.

[B20] Limesurvey GmbH (2020). LimeSurvey: An Open Source Survey Tool. Hamburg: LimeSurvey GmbH. Available online at: http://www.limesurvey.org

[B21] Little T. D. and Kline, R. B. (eds). (2016). Principles and Practice of Structural Equation Modeling, 4th ed. New York, NY: The Guilford Press

[B22] Liu I. F. Chen M. C. Sun Y. S. Wible D. Kuo C. H. (2010). Extending the TAM model to explore the factors that affect intention to use an online learning community. Comput. Educ. 54, 600–610. 10.1016/j.compedu.2009.09.009

[B23] Moon J. W. Kim Y. G. (2001). Extending the TAM for a World-Wide-Web context. Inf. Manage. 38, 217–230. 10.1016/S0378-7206(00)00061-6

[B24] Persico D. Manca S. Pozzi F. (2014). Adapting the technology acceptance model to evaluate the innovative potential of e-learning systems. Comput. Human Behav. 30, 614–622. 10.1016/j.chb.2013.07.045

[B25] Rauniar R. Rawski G. Yang J. Johnson B. (2014). Technology acceptance model (TAM) and social media usage: an empirical study on Facebook. J. Enterprise Inf. Manage. 27, 6–30. 10.1108/JEIM-04-2012-0011

[B26] Reuterberg S. E. Gustafsson J. E. (1992). Confirmatory factor analysis and reliability: testing measurement model assumptions. Educ. Psychol. Meas. 52, 795–811. 10.1177/0013164492052004001

[B27] Sass D. A. Smith P. L. (2006). The effects of parceling unidimensional scales on structural parameter estimates in structural equation modeling. Struct. Equ. Model. 13, 566–586. 10.1207/s15328007sem1304_4

[B28] Schermelleh-Engel K. Moosbrugger H. (2003). Evaluating the fit of structural equation models: tests of significance and descriptive goodness-of-fit measures. Methods Psychol. Res. 8, 23–74. Available online at: https://www.researchgate.net/publication/251060246_Evaluating_the_Fit_of_Structural_Equation_Models_Tests_of_Significance_and_Descriptive_Goodness-of-Fit_Measures

[B29] Servidio R. Cronin M. (2018). PerLE: an ‘open-source', e-learning moodle-based platform. A study of university undergraduates acceptance. Behav. Sci. 8:63. 10.3390/bs8070063 30012998 PMC6070931

[B30] Shin W. S. Kang M. (2015). The use of a mobile learning management system at an online university and its effect on learning satisfaction and achievement. Int. Rev. Res. Open Distrib. Learn. 16, 110–130. 10.19173/irrodl.v16i3.1984

[B31] Steyer R. Eid M. (2002). Messen und Testen. Berlin: Springer. 10.1007/978-3-642-56924-1

[B32] Sumak B. Hericko M. Pusnik M. (2011). A meta-analysis of e-learning technology acceptance: the role of user types and e-learning technology types. Comput. Human Behav. 27, 2067–2077. 10.1016/j.chb.2011.08.005

[B33] Sun H. Zhang P. (2006). The role of moderating factors in user technology acceptance. Int. J. Hum. Comput. Stud. 64, 53–78. 10.1016/j.ijhcs.2005.04.01327564428

[B34] Venkatesh V. (2000). Determinants of perceived ease of use: integrating control, intrinsic motivation, and emotion into the technology acceptance model. Inf. Syst. Res. 11, 342–365. 10.1287/isre.11.4.342.11872

[B35] Venkatesh V. Davis D. F. (1996). A model of the antecendents of perceived ease of use: development and test. Dec. Sci. 27, 451–481. 10.1111/j.1540-5915.1996.tb01822.x

[B36] Venkatesh V. Davis F. D. (2000). A theoretical extension of the technology acceptance model. Four longitudinal field studies. Manage. Sci. 46, 186–204. 10.1287/mnsc.46.2.186.11926

[B37] Wong K. T. Osman R. Goh P. S. C. Rahmat M. K. (2013). Understanding student teachers' behavioural intention to use technology: technology acceptance model (TAM) validation and testing. Int. J. Instruct. 6, 89–104 Available online at: https://www.e-iji.net/dosyalar/iji_2013_1_7.pdf

[B38] Yeou M. (2016). An investigation of students acceptance of moodle in a blended learning setting using technology acceptance model. J. Educ. Technol. Syst. 44, 300–318. 10.1177/0047239515618464

[B39] Yousafzai S. Y. Foxall G. R. Paliister J. G. (2007). Technology acceptance: a meta-analysis of the TAM: Part 1. J. Model. Manag. 2, 251–280. 10.1108/17465660710834453

