# Supplementary material for: An Evaluation of Forced Distance Learning and Teaching Under Pandemic Conditions Using the Technology Acceptance Model
Source: Front Psychol. 2021 Oct 21;12:701347. doi: 10.3389/fpsyg.2021.701347 (PMC8572054; doi:10.3389/fpsyg.2021.701347)
Supplement: Supplementary file 1 [file Table_2.docx]

**Supplementary Table 2:** Items of the FDL and FDT-subscales, standardized factor loadings (FL) and indicator reliabilities (IR) (students) of the items which were included into the path models

| **Scale^1^** | **No.** | **STUDENTS** | | | | **LECTURERS** | |
| --- | --- | --- | --- | --- | --- | --- | --- |
|  |  | **German** | **English** | **FL^2^** | **IR^3^** | **German** | **English** |
| **PU** | 1 | Die Nutzung der Live-Streams / Videoaufzeichnungen erleichtert mir das Lernen. | Using the live streams / video recordings makes learning easier for me. | .90 | .81 | Anhand von Live-Streams / Videoaufzeichnungen kann ich Lerninhalte gut vermitteln. | By using live streams / video recordings I can teach learning contents well. |
|  | 2 | Durch die Nutzung der Live-Streams / Videoaufzeichnungen kann ich neue Lerninhalte besser verstehen. | By using the live streams / video recordings I understand new learning content better. | .83 | .70 |  |  |
|  | 3 | Durch die Nutzung der Live-Streams / Videoaufzeichnungen setze ich mich mit den Inhalten der Lehrveranstaltung auseinander. | By using the live streams / video recordings, I deal with the contents of the course. |  |  | Durch die Bereitstellung von Live-Streams / Videoaufzeichnungen setzen sich die Studierenden mit den Inhalten meiner Lehrveranstaltung auseinander. | By providing live streams / video recordings the students deal with the contents of my course. |
|  | 4 | Durch die Nutzung der Live-Streams / Videoaufzeichnungen kann ich mich gut auf Prüfungen vorbereiten. | By using the live streams / video recordings I prepare well for exams. | .84 | .71 | Anhand von Live-Streams / Videoaufzeichnungen kann ich die Studierenden auf Prüfungen vorbereiten. | By using live streams / video recordings I train the students for their exams. |
| **PEOU** | 5 | Der Umgang mit den Live-Streams / Videoaufzeichnungen ist für mich klar und verständlich. | The usage of the live streams / video recordings is obvious and understandable for me. | .76 | .58 | Für mich ist klar und verständlich, wie ich Live-Streams durchführen / Videoaufzeichnungen erstellen kann. | It is obvious and understandable for me how I can create live streams / video recordings. |
|  | 6 | Der Einarbeitungsaufwand zur Nutzung der Live-Streams / Videoaufzeichnungen war gering. | The training effort to use the live streams / video recordings was low. | .70 | .49 | Der Einarbeitungsaufwand in die Durchführung von Live-Streams / das Erstellen von Videoaufzeichnungen war / ist gering. | The training effort for the execution of live streams / the creation of video recordings was / is low. |
| **ATU** | 7 | Ich bin mit den Live-Streams / Videoaufzeichnungen zufrieden. | I am satisfied with the live streams / video recordings. | .81 | .66 | Ich bin mit den Möglichkeiten zufrieden, die ich durch Live-Streams / Videoaufzeichnungen in meiner Lehre habe. | I am satisfied with the possibilities I have in my teaching through live streams / video recordings. |
|  | 8 | Ich würde die Live-Streams / Videoaufzeichnungen weiterempfehlen. | I would recommend the live streams / video recordings. | .82 | .85 | Die Lehre mit Live-Streams / Videoaufzeichnungen würde ich Kollegen weiterempfehlen. | I would recommend teaching with live streams / video recordings to colleagues. |
|  | 9 | Die Live-Streams / Videoaufzeichnungen sind ein geeigneter Ersatz für Präsenzveranstaltungen. | The live streams / video recordings are a convenient substitute for face-to-face events. | .70 | .50 | Live-Streams / Videoaufzeichnungen sind ein geeigneter Ersatz für meine Präsenzveranstaltungen. | Live streams / video recordings are a convenient substitute for my face-to-face events. |
|  | 10 | Ich würde mir auch zukünftig Live-Streams / Videoaufzeichnungen wünschen. | I would also like to have live streams / video recordings in the future. | .78 | .61 | Ich würde gerne auch zukünftig anhand von Live-Streams / Videoaufzeichnungen unterrichten. | I would like to continue teaching in the future using live streams / video recordings. |
| **AU** | 11 | Ich nutze die Live-Streams / Videoaufzeichnungen, die für mein Semester angeboten werden. | I use the live streams / video recordings that are offered for my semester. | .80 | .80 | Ich biete alle meine Lehrveranstaltungen als Live-Streams / Videoaufzeichnungen an. | I offer all of my courses as live streams / video recordings. |
|  | 12 | Ich informiere mich regelmäßig, ob neue Live-Streams / Videoaufzeichnungen zur Verfügung stehen. | I check regularly if new live streams / video recordings are available. | .33 | .33 | Ich habe mich informiert, welches Lehrangebot ich mit Hilfe von Live-Streams / Videoaufzeichnungen anbieten kann. | I have researched which teaching services I can offer with the help of live streams / video recordings. |
|  | 13 |  |  |  |  | Ich habe mich mit den technischen Möglichkeiten (z.B. Breakout sessions, Umfragen, Chat) der Live-Streams / Videoaufzeichnungen auseinandergesetzt. | I have explored the technical possibilities (e.g. breakout sessions, surveys, chat) of live streams / video recordings. |
|  | 14 | Ich nutze die Live-Streams / Videoaufzeichnungen intensiv zum Lernen. | I use the live streams / video recordings intensively for learning. |  |  |  |  |
| **GMA** | 15 | Ich finde mich schnell in neuer Software zurecht. | I quickly find my way around new software. | .90 | .81 | Ich finde mich schnell in neuer Software zurecht. | I quickly find my way around new software. |
|  | 16 | Ich schätze mich als erfahrene(n) Computernutzer/in ein. | I consider myself an experienced computer user. | .70 | .48 | Ich schätze mich als erfahrene(n) Computernutzer/in ein. | I consider myself an experienced computer user. |
|  | 17 | Ich habe bereits vor der derzeitigen Situation Videokonferenzprogramme verwendet. | I have been already using video conferencing programs before the current situation. |  |  | Ich habe bereits vor der derzeitigen Situation Videokonferenzprogramme verwendet. | I have been already using video conferencing programs before the current situation. |
| **DSW** | 18 | Ich habe Angst, dass es bei der Nutzung der Videokonferenzprogramme zu einem Datenmissbrauch kommt. | I am concerned that data misuse may occur when using the video conferencing programs. | .93 | .87 | Ich habe Angst, dass es durch die Nutzung der Videokonferenzprogramme zu einem Datenmissbrauch kommt. | I am concerned that the use of video conferencing programs may lead to data misuse. |
|  | 19 | Ich habe Angst, während der Nutzung der Videokonferenzprogramme ausspioniert zu werden. | I am concerned about being spied on while using the video conferencing programs. | .83 | .68 | Ich habe Angst, während der Nutzung der Videokonferenzprogramme ausspioniert zu werden. | I am concerned about being spied on while using the video conferencing programs. |
|  | 20 |  |  |  |  | Ich habe Angst, dass meine Videoaufzeichnungen unberechtigterweise weiter verbreitet werden. | I am concerned that my video recordings will be distributed without authorization. |
| **PW** | 21 | Ich fühle mich durch die Corona-Pandemie belastet. | I feel negatively affected by the Corona pandemic. | .83 | .29 | Ich fühle mich durch die Corona-Pandemie belastet. | I feel negatively affected by the Corona pandemic. |
|  | 22 | Ich habe die Sorge, dass mir trotz der Nutzung der Live-Streams / Videoaufzeichnungen Wissenslücken entstehen, die ich nicht schließen kann. | I am concerned that despite the use of the live streams / video recordings, knowledge deficits arise which I cannot handle. | .51 | .68 |  |  |
|  | 23 | Das Studieren bzw. Arbeiten ist schwierig für mich, da ich keinen geeigneten Ort für die Nutzung der Live-Streams / Videoaufzeichnungen habe. | Studying is difficult for me because I do not have a suitable place to use the live streams / video recordings. | .54 | .26 |  |  |
|  | 24 | Ich habe finanzielle Sorgen (z.B. durch Verdienstverlust, Zusatzkosten durch Internet, technische Ausrüstung oder Anschaffungen). | I experience financial difficulties (e.g. loss of earnings, additional costs due to internet, technical equipment or purchases). |  |  |  |  |
| **TI** | 25 | Ich verfüge über eine geeignete technische Ausrüstung zur Nutzung der Live-Streams / Videoaufzeichnungen. | I have suitable technical equipment to use the live streams / video recordings. | .65 | .42 | Ich verfüge über eine geeignete technische Ausrüstung für die Durchführung von Live-Streams / das Erstellen von Videoaufzeichnungen. | I have a suitable technical equipment for live streaming / video recording. |
|  | 26 | Die Qualität (z.B. Geschwindigkeit, Stabilität) der Internetverbindung zur Nutzung der Live-Streams ist gut. | The quality (e.g. speed, stability) of the Internet connection for using the live streams is good. | .66 | .44 | Die Qualität (z.B. Geschwindigkeit, Stabilität) der Internetverbindung für Live-Streams ist gut. | The quality (e.g. speed, stability) of the Internet connection for live streams is good. |
|  | 27 | Bei technischen Fragen habe ich einen Ansprechpartner. | For technical questions I have an appropriate contact person. |  |  | Bei technischen Fragen habe ich einen Ansprechpartner. | For technical questions I have an appropriate contact person. |
| **OT** | 28 | Die Lehr- und Lernmaterialien standen bzw. stehen rechtzeitig zur Verfügung. | The teaching and learning materials were or are available in time. | .64 | .42 |  |  |
|  | 29 | Die Lehr- und Lernmaterialien sind leicht zu finden. | The teaching and learning materials are easy to find. | .65 | .43 |  |  |
|  | 30 | Es war bzw. ist rechtzeitig bekannt, wann welche Live-Streams stattfinden. | It was or is known in sufficient time when which live streams will take place. | .50 | .25 |  |  |
|  | 31 | Bei inhaltlichen Fragen habe ich eine Ansprechperson. | I have a contact person for content-related questions. | .50 | .25 |  |  |

^1^Scale: PU = Perceived usefulness; PEOU = Perceived ease of use; ATU = Attitude towards using; AU = Actual system use; GMA = General media affinity; DSW = Data security worries; PW = Pandemic related worries; TI = Technical infrastructure; OT = Organization of online teaching; ^2^FL = standardized factor loadings; ^3^IR = Indicator reliability; items marked in grey were not included into the final model
